# A User’s View of the Parameters Derived from the Induction Curves of Maximal Chlorophyll *a* Fluorescence: Perspectives for Analyzing Stress

**DOI:** 10.3389/fpls.2016.01679

**Published:** 2016-11-11

**Authors:** Julie Ripoll, Nadia Bertin, Luc P. R. Bidel, Laurent Urban

**Affiliations:** ^1^INRA – Centre d’Avignon, UR 1115 Plantes et Systèmes de Culture HorticolesAvignon, France; ^2^UMR QualiSud, Université d’Avignon et des Pays du VaucluseAvignon, France; ^3^INRA, UMR AGAPMontpellier, France

**Keywords:** chlorophyll *a* fluorescence, JIP-test, potential and limits, *Solanum lycopersicum* L., stress response, water deficit

## Abstract

Analysis of the fast kinetics of the induction curve of maximal fluorescence represents a relatively recent development for chlorophyll *a* fluorescence measurements. The parameters of the so-called JIP-test are exploited by an increasingly large community of users to assess plant stress and its consequences. We provide here evidence that these parameters are capable to distinguish between stresses of different natures or intensities, and between stressed plants of different genetic background or at different developmental stages at the time of stress. It is, however, important to keep in mind that the JIP-test is inherently limited in scope, that it is based on assumptions which are not fully validated and that precautions must be taken to ensure that measurements are meaningful. Recent advances suggest that some improvements could be implemented to increase the reliability of measurements and the pertinence of the parameters calculated. We moreover advocate for using the JIP-test in combination with other techniques to build comprehensive pictures of plant responses to stress.

## Introduction

There is a growing interest for ChlF since the last 15 years (e.g., 14400 articles in 2015 vs. 4870 in 2000; research made on google scholar using “chlorophyll *a* fluorescence” as a keyword). When excitation energy arrives in a RC where the donor side cannot evacuate energy toward an acceptor, energy is essentially lost under the form of heat and ChlF. Measurements of ChlF therefore gives insight into efficiencies of energy transfer and heat dissipation. Stress may impact all the steps from light energy absorption to electron transfer to the final acceptors. So, ChlF can be used to characterize the effects of stress on adaptive mechanisms (Misra et al., 2012; Kalaji et al., 2014, 2016).

There are several types of instruments for analyzing ChlF. Steady-state instruments are designed for quenching analysis and for coupled measurements of ChlF and gas exchanges which give insight into downstream processes. The focus here is on the analysis of OJIP fluorescence transients (JIP-test) which become increasingly popular among users, thanks to the development of relatively cheap and users-friendly devices (Kalaji et al., 2014). In this paper, we shall exploit the experience we have gained using the JIP-test for evaluating the effects of different intensities of WD on tomato plants according to genetic diversity and to plant developmental stage at the time of stress, to discuss its potential. We shall also stress some theoretical and practical limitations of the JIP-test and evoke its potential when combined to other techniques.

## The Ojip/Olkjip Model: Principle and Short Description

The OJIP model allows to analyze the ChlF induction curve when a leaf acclimated to dark conditions is suddenly exposed to a saturating pulse of light (Kautsky effect). The induction curve appears as a fast wave (ca. 0.3 s) with characteristic steps named O, J, I, and P, plotted on a logarithmic time scale, starting from initial fluorescence *F*_0_ (dark adapted) to maximal fluorescence *F*_M_ (light-saturated; Strasser and Strasser, 1995). Level O corresponds to the initial fluorescence emitted, whereas levels J and I correspond to the fluorescence emitted after, respectively, 2 and 30 ms. The level P corresponds to *F*_M_ (Strasser et al., 2000). Under specific conditions, like heat stress, another inflection in the induction curve can appear around 300 μs, called K. Eventually a shift of the induction curve between 50 and 300 μs, influenced by the excitation energy transfer between PS II units, may appear, the so-called L band (Strasser and Stirbet, 1998).

The JIP-test is based on several assumptions (Stirbet and Govindjee, 2011). The most important assumption is that the fluorescence increase from *F*_0_ to *F*_M_ reflects mainly the redox state of Q_A_ protein (Kalaji et al., 2014) in PSII RC. This basic assumption is a matter of debate (Schansker et al., 2014). For some authors, the alterations induced at the acceptor side of PSII RC during the rapid turnover of oxidation of the PQ pool at the Q_B_ site may be essential in the triggering of photoinactivation and D1 protein damage (Gong and Ohad, 1991). Within this view it is the Q_B_ state occupancy which has the highest influence on fluorescence yield (Zivčák et al., 2015). The JIP-test is not only based on the assumption that the *F*_0_ to *F*_M_ rise reflects the Q_A_ redox state, but also on the assumption that NPQ processes do not hinder the rise to *F*_M_.

The JIP-test can be used as a signature of (1) diverse events translating into changes in the redox state of the components of the linear electron transport flow, (2) the involvement of alternative electron routes, (3) the build-up of a transmembrane pH gradient (and membrane potential), (4) the activation of different NPQ processes, (5) the activation of the Calvin-Benson cycle (Stirbet et al., 2014). The OJ section reflects the reduction of the acceptor side of PSII, the JI section the partial reduction of the PQ pool and finally the IP section the reduction of the acceptor side of PSI. The reader will find an excellent introduction to the parameters derived from the JIP-test mathematical model in Strasser et al. (2004) and Stirbet and Govindjee (2011) for instance. The key idea underpinning stress characterization and analysis using the JIP-test, is that stress necessarily impacts the efficiencies and fluxes of electrons and of energy in and around PSI and PSII, and that their variations can be assessed and analyzed using the parameters derived from the OJIP/OLKJIP transients (Maxwell and Johnson, 2000).

## What Can we Learn from the Parameters of the Jip-Test?

**FIGURE 1:**
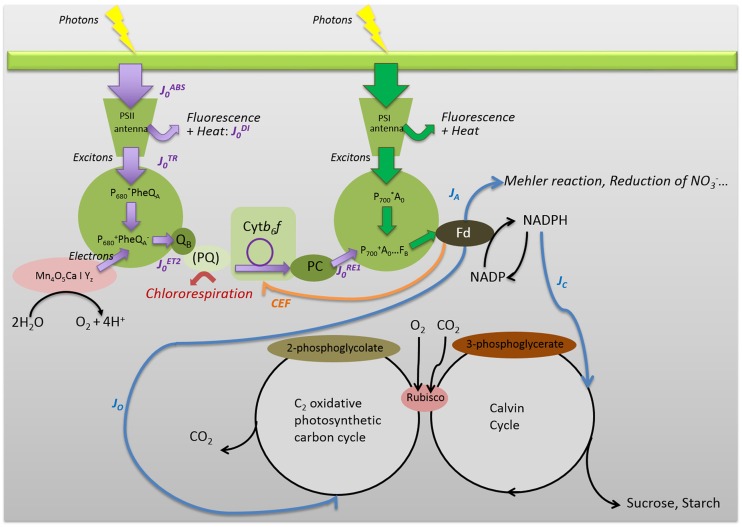
**Simplified representation of the main energy pathways in and around PSI and PSII, down to ferredoxin (Fd) and downstream.** Some of the JIP-test parameters were indicated. Since most ChlF originates in PSII antenna, J_0_^ABS^ represents the rate of photon absorption by all PSII antenna pigments. The dissipated energy flux J_0_^DI^ represents the part of the absorbed photon flux dissipated through direct fluorescence and other non-radiative processes (as heat), and the trapped exciton flux J_0_^TR^ represents the rate of exciton trapping by the PSII RC P_680_. The trapped energy is used for charge separation using the components of the PSII RC, i.e., pheophytin molecules (Phe) and Q_A_ and Q_B_ quinones [linked to D1 and D2 proteins not represented, Q_A_ (bound to D2) and Q_B_ (bound to D1)]. The complex Mn_4_O_5_Ca|Y_2_ corresponds to the oxygen-evolving complex. The flux J_0_^ET2^ represents the electron transport flux from Q_A_ to Q_B_. J_0_^RE1^ represents the rate of electrons from Q_B_ to PSI acceptors. J_C_, J_O,_ and J_A_ represent the electron flows for carboxylation, oxygenation, and alternative sinks, respectively. PQ and PC represent plastoquinons and plastocyanins. Violet arrows are associated to the JIP-test. Blue arrows correspond to the fluxes evaluated from combined measurements of modulated ChlF and gas exchanges (Valentini et al., 1995). The orange arrow corresponds to the CEF (Kotakis et al., 2006) and the red to the chlororespiration (Rumeau et al., 2007).

Plants have to adapt to the risk of photooxidative damage which results from the imbalance between the incoming energy under the form of photon flux and the energy quenched by photosynthetic processes (Gururani et al., 2015). Stress, for instance by limiting stomatal conductance and CO_2_ supply to the Calvin–Benson cycle, exacerbates the risk of photooxidative damage. Therefore, stress triggers adaptive responses aiming at reducing the quantity of energy entering the leaf, reducing the amount of absorbed energy converted into electron flux, and rerouting electron fluxes. Each step of OJIP/OKJIP curves can be associated to the efficiency of energy or electron transfers between the components of PSII and PSI (**Figure 1**).

The JIP-test provides invaluable parameters to analyze upstream adaptive mechanisms to different types of stress (Kalaji et al., 2016). It is, however, important to keep in mind that no single parameter derived from the JIP-test can be considered as specific of a given type of stress. It is rather a combination of parameters that may be considered as relevant. The rate of energy dissipation by processes other than trapping expressed either per RCs or on an absorbed energy basis J_0_^DI^/RC or J_0_^DI^/J^ABS^ is used to evaluate heat dissipation processes. The ratio V _K_/V _J_[= (F_0.3ms_ - F_0_)/(F_2ms_ - F_0_)] is associated to limitation/inactivation and possibly damage of the oxygen-evolving complex. The I-P phase, consequently the rate of electron transport from Q_B_ to PSI acceptors J_0_^RE1^/RC or J_0_^RE1^/J^ABS^ is considered to give insight into the CEF (Harbinson and Foyer, 1991; Schansker et al., 2014; Zivčák et al., 2015). The CEF contributes to the balance of the ATP/NADPH output ratio and can provide protection against photooxidative stress (Martin et al., 2004; Huang et al., 2016), offsetting the decline of the linear electron flow under WD (Mladenov et al., 2015).

There is an increasing amount of evidence suggesting that the JIP-test is a discriminating one. The JIP-test is capable not only to assess different kind of stresses but also to distinguish between specific responses for a given type of stress, due, for instance, to genotypic differences, to differences in stress intensity, or to differences in the developmental stage of plants at the time stress is applied. JIP parameters were found to be capable to distinguish among tree species (Pollastrini et al., 2016). The above-mentioned parameters, J_0_^DI^/J^ABS^, V _K_/V _J_ and J_0_^RE1^/J^ABS^, were found to be relevant parameters to evaluate responses to WD as a function of genotype diversity for a given plant species, in barley (Oukarroum et al., 2007, 2009) and in tomato (**Table 1**). Considering differences according to stress intensity, we observed a difference in J_0_^RE1^/J^ABS^ between tomato plants at the reproductive stage submitted to severe WD and tomato plants at the same stage of development submitted to repeated cycles of WD and recovery (**Table 1**). We also found differences due to developmental stage at the time of stress since we observed an increase in J_0_^DI^/RC and J_0_^DI^/J^ABS^ in tomato plants at the reproductive stage submitted to severe WD (with the exception of LA1420), whereas there was an increase in *F*_0_ and a decrease in *S*_m_ (the normalized area of the maximal fluorescence induction curve) in plants at the vegetative stage submitted to a similar stress (**Table 1**). The latter shifts are suspected to be indicators of damage (Christen et al., 2007; Yordanov et al., 2008).

**Table 1:** Relative differences for the parameters derived from JIP-test, performed on 30 min dark-adapted leaves with a Plant Efficiency Analyzer (Hansatech Instrument, King’s Lynn, UK) on vegetative and reproductive plants of Cervil, LA1420, PlovdivXXIVa and Levovil tomato accessions exposed to two WD treatments.

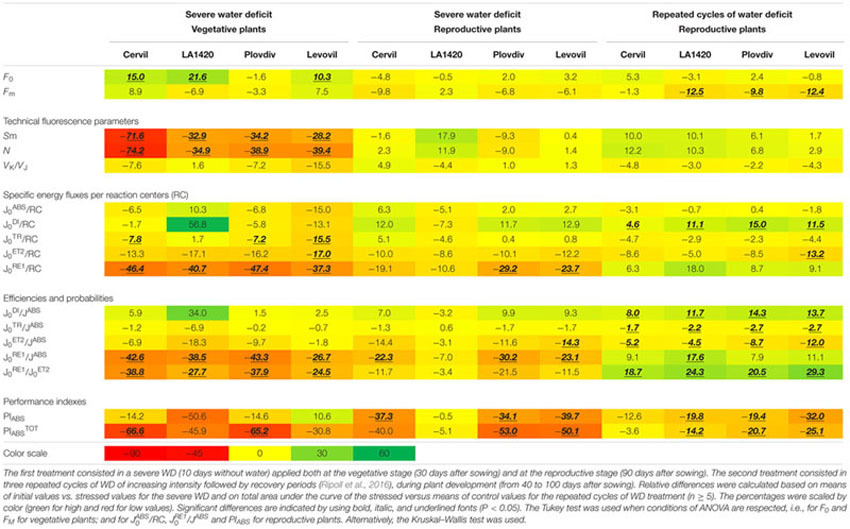

It is near to impossible not to evoke the popular Performance Index (PI) of Strasser when discussing the parameters derived from the JIP-test (Silvestre et al., 2014; Zivčák et al., 2014). Recently Kalaji et al. (2016) recommended the non-specialist to resort to the PI in the absence of a serious capacity to understand and exploit the other parameters. We experienced that the PI is not always as easy to interpret as usually believed. For instance, the 19.8% decrease in PI_ABS_ of LA1420 plants at the reproductive stage submitted to repeated cycles of WD and recovery (generally believed to favor acclimation) withstands interpretation since PI_ABS_ did not decrease in similar plants submitted to severe WD (**Table 1**). One would have expected the reverse. Our opinion is that it is generally more rewarding to make use of the full set of parameters that can be derived from the JIP-test.

## Limitations Originating from the Theoretical Background, Recent Advances and Prospects Offered by Combining Approaches

There are several limitations associated with the JIP-test, some arising from the physiological assumptions behind the theory and others concerning good practices (Murchie and Lawson, 2013; Kalaji et al., 2014). As said before, it is essential to keep in mind that the JIP-test model is based on a sum of assumptions (Stirbet and Govindjee, 2011). For instance, during the measurement of an OJIP transient, all PSII units are considered to be homogeneous and active, which is probably not true (Vredenberg, 2011). Recent mathematical models using KMC simulation can help to deal with this limitation (Guo and Tan, 2011, 2014). KMC simulation should help to take into account the variability in the number of RCs, in PQ pool size, in the number of active Q_B_ sites and in Q_A_ reduction rate events (Guo and Tan, 2011, 2014).

However, the information supplied by JIP parameters does not allow for comprehensive interpretation of the adaptive strategies adopted by stressed plants. This is a shortcoming inherent to the fact that all the information derived from the JIP-test is about energy and electron fluxes and transfer efficiencies upstream PSI, whereas it is quite clear that downstream allocation of electron fluxes among the Calvin-Benson cycle, photorespiration and alternative electron sinks play a key-role along with antioxidant mechanisms in the strategy of plants facing photooxidative damage (**Figure 1**). The fluorescence steps beyond *F*_M_ so-called PSMT phase (Kalaji et al., 2016) could be used for analyses in relation to the activation of the ferredoxin-NADP+ reductase and the Calvin–Benson cycle through the ferredoxin-thioredoxin system (Stirbet et al., 2014). So far, unfortunately, the PSMT phase appears less reproducible than the OJIP phase (Stirbet et al., 2014; Vredenberg, 2015).

Recent studies bridged the gap between the scientific sub-communities by associating analysis of the OJIP transients, measurements of gas exchanges and simultaneous measurements of PSI and PSII activities, with the objective to characterize PSI functioning (Brestič et al., 2014; Zivčák et al., 2015). Such approaches should be more developed in the future to build broader pictures of the mechanisms of plant acclimation to stress at play both before and beyond PSI. The information obtained could possibly be used to improve the PSMT model and to gain new insight in the functioning of the components of the photosynthetic machinery (Belyaeva et al., 2016). Of course there is also ample room for progress by studying jointly parameters derived from ChlF measurements and molecular and biochemical markers (Hao et al., 2012; Mladenov et al., 2015; Yin et al., 2015).

## Concluding Remarks

JIP parameters are gaining recognition among plant biologists besides other indicators of physiological status (Chen et al., 2014; Wituszyńska et al., 2015). There is little doubt that improvements and novel techniques like JIP-test imaging (Jedmowski and Brüggemann, 2015) will go on fueling the interest of the scientific community for these parameters in the future, possibly in phenotyping platforms. The potential of JIP parameters to distinguish between plant stress responses and to assess genetic diversity is more and more well recognized. However, for interesting they are, the parameters derived from the JIP-test have inherent limitations. We therefore recommend to associate to JIP parameters to parameters derived from combined measurements of gas-exchanges and steady-state ChlF, and even to other molecular or biochemical markers, to get the most comprehensive pictures possible of the plant adaptive mechanisms involved in stress responses.

## Author Contributions

JR, NB, LB, and LU compiled data, developed theory and wrote the paper. JR performed the experiments and analyses.

## Conflict of Interest Statement

The authors declare that the research was conducted in the absence of any commercial or financial relationships that could be construed as a potential conflict of interest.

## Abbreviations

CEF: cyclic electron flux
ChlF: chlorophyll *a* fluorescence
KMC: Kinetic Monte Carlo
NPQ: non-photochemical quenching
PC: plastocyanin
PQ: plastoquinone
PS: photosystem
RC: reaction center
WD: water deficit.
